# 1,4-Dibromo­butane-2,3-dione

**DOI:** 10.1107/S1600536812044200

**Published:** 2012-10-31

**Authors:** De-Xin Zai

**Affiliations:** a College of Science, Nanjing Forestry University, No. 159, Longpan Road, Nanjing 210037, People’s Republic of China

## Abstract

The asymmetric unit of the title compound, C_4_H_4_Br_2_O_2_, contains one half-mol­ecule, being located about a centre of inversion. In the crystal, there are no significant inter­molecular inter­actions.

## Related literature

For the uses of 1,4-dibromo­butane-2,3-dione, see: Gogte *et al.* (1967 ▶). For the synthesis of 1,4-dibromo­butane-2,3-dione, see: Ruggli & Herzog (1946 ▶). For the cystal structure of the 1,4-di­chloro analogue, see: Ducourant *et al.* (1986 ▶). For bond–length data, see: Allen *et al.* (1987 ▶).
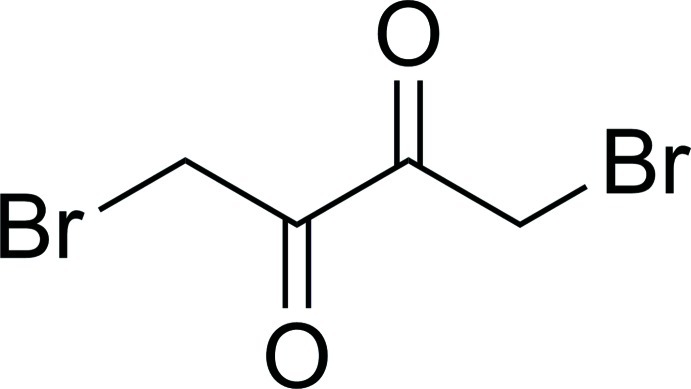


## Experimental

### 

#### Crystal data

C_4_H_4_Br_2_O_2_
*M* *_r_* = 243.89
Orthorhombic, 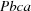

*a* = 6.945 (1) Å
*b* = 5.542 (1) Å
*c* = 17.238 (3) Å
*V* = 663.5 (2) Å^3^
*Z* = 4
Mo *K*α radiation
μ = 12.13 mm^−1^
*T* = 298 K
0.10 × 0.10 × 0.10 mm

#### Data collection

Enraf–Nonius CAD-4 diffractometer
Absorption correction: ψ scan (North *et al.*, 1968 ▶) *T*_min_ = 0.195, *T*_max_ = 0.377
614 measured reflections
614 independent reflections
319 reflections with *I* > 2σ(*I*)
*R*_int_ = 0.077
3 standard reflections every 120 min intensity decay: 1%

#### Refinement

*R*[*F* ^2^ > 2σ(*F* ^2^)] = 0.065
*wR*(*F* ^2^) = 0.108
*S* = 0.93
614 reflections
37 parameters
1 restraint
H-atom parameters constrained
Δρ_max_ = 0.65 e Å^−3^
Δρ_min_ = −0.60 e Å^−3^

### 

Data collection: *CAD-4 Software* (Enraf–Nonius, 1985 ▶); cell refinement: *CAD-4 Software*; data reduction: *XCAD4* (Harms & Wocadlo, 1995 ▶); program(s) used to solve structure: *SHELXS97* (Sheldrick, 2008 ▶); program(s) used to refine structure: *SHELXL97* (Sheldrick, 2008 ▶); molecular graphics: *SHELXTL* (Sheldrick, 2008 ▶); software used to prepare material for publication: *SHELXTL*.

## Supplementary Material

Crystal structure: contains datablock(s) I, global, n1. DOI: 10.1107/S1600536812044200/su2517sup1.cif

Structure factors: contains datablock(s) I. DOI: 10.1107/S1600536812044200/su2517Isup2.hkl

Supplementary material file. DOI: 10.1107/S1600536812044200/su2517Isup3.cml

Additional supplementary materials:  crystallographic information; 3D view; checkCIF report

## Figures and Tables

**Fig. 1.**
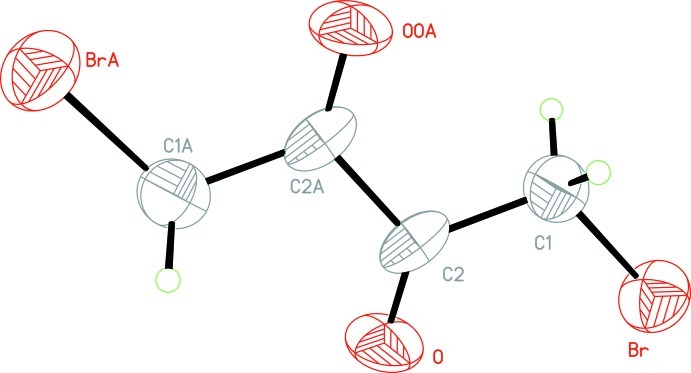
The molecular structure of the title molecule, with the atom numbering. Displacement ellipsoids are drawn at the 50% probability level [Symmetry code: (a) -x+2, -y+1, -z+1].

**Fig. 2.**
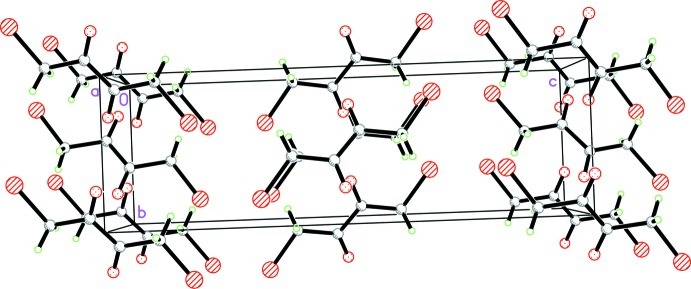
A view along the a axis of the crystal packing of the title compound.

## supplementary crystallographic information

### Comment

1,4-Dibromobutane-2,3-dione and its derivatives are important intermediates for the synthesis of compounds possessing promising anticancer activity, which is attributed to their likely interference in the hexose-monophosphate (HMP) pathway (Gogte *et al.*, 1967). We report herein on the crystal structure of the title compound.

In the title molecule, Fig. 1, the bond lengths (Allen *et al.*, 1987) and angles are within normal ranges. The asymmetric unit contains one half-molecule, being located on a centre of inversion. It can be compared to the 1,4-dichloro derivative that crystallized in the monoclinic space group P2_1_/c (Ducourant *et al.*, 1986) but which also possesses inversion symmetry.

In the crystal, there are no significant intermolecular interactions (Fig. 2).

### Experimental

1,4-Dibromobutane-2,3-dione was prepared by the method reported in the literature (Ruggli & Herzog, 1946). Yellow plate-like crystals were obtained by dissolving the title compound (0.50 g, 2.05 mmol) in dichloromethane (30 ml) and evaporating the solvent slowly at room temperature for ca. 2 days.

### Refinement

The methylene H atoms were positioned geometrically and refined as riding atoms: C-H = 0.97 Å, with U_iso_(H) = 1.2U_eq_(C).

### Crystal data

**Table:**

C_4_H_4_Br_2_O_2_	*D*_x_ = 2.442 Mg m^−^^3^
*M**_r_* = 243.89	Melting point < 395 K
Orthorhombic, *P**b**c**a*	Mo *K*α radiation, λ = 0.71073 Å
Hall symbol: -P 2ac 2ab	Cell parameters from 25 reflections
*a* = 6.945 (1) Å	θ = 9–14°
*b* = 5.542 (1) Å	µ = 12.13 mm^−^^1^
*c* = 17.238 (3) Å	*T* = 298 K
*V* = 663.5 (2) Å^3^	Cube, yellow
*Z* = 4	0.10 × 0.10 × 0.10 mm
*F*(000) = 456	

### Data collection

**Table:**

Enraf–Nonius CAD-4 diffractometer	319 reflections with *I* > 2σ(*I*)
Radiation source: fine-focus sealed tube	*R*_int_ = 0.077
Graphite monochromator	θ_max_ = 25.4°, θ_min_ = 2.4°
ω/2θ scans	*h* = 0→8
Absorption correction: ψ scan (North *et al.*, 1968)	*k* = −6→6
*T*_min_ = 0.195, *T*_max_ = 0.377	*l* = −20→20
614 measured reflections	3 standard reflections every 120 min
614 independent reflections	intensity decay: 1%

### Refinement

**Table:**

Refinement on *F*^2^	Primary atom site location: structure-invariant direct methods
Least-squares matrix: full	Secondary atom site location: difference Fourier map
*R*[*F*^2^ > 2σ(*F*^2^)] = 0.065	Hydrogen site location: inferred from neighbouring sites
*wR*(*F*^2^) = 0.108	H-atom parameters constrained
*S* = 0.93	*w* = 1/[σ^2^(*F*_o_^2^) + (0.*P*)^2^] where *P* = (*F*_o_^2^ + 2*F*_c_^2^)/3
614 reflections	(Δ/σ)_max_ < 0.001
37 parameters	Δρ_max_ = 0.65 e Å^−^^3^
1 restraint	Δρ_min_ = −0.60 e Å^−^^3^

### Special details

**Table:**

Geometry. All e.s.d.'s (except the e.s.d. in the dihedral angle between two l.s. planes) are estimated using the full covariance matrix. The cell e.s.d.'s are taken into account individually in the estimation of e.s.d.'s in distances, angles and torsion angles; correlations between e.s.d.'s in cell parameters are only used when they are defined by crystal symmetry. An approximate (isotropic) treatment of cell e.s.d.'s is used for estimating e.s.d.'s involving l.s. planes.
Refinement. Refinement of *F*^2^ against ALL reflections. The weighted *R*-factor *wR* and goodness of fit *S* are based on *F*^2^, conventional *R*-factors *R* are based on *F*, with *F* set to zero for negative *F*^2^. The threshold expression of *F*^2^ > σ(*F*^2^) is used only for calculating *R*-factors(gt) *etc*. and is not relevant to the choice of reflections for refinement.

### Fractional atomic coordinates and isotropic or equivalent isotropic displacement parameters (Å^2^)

**Table:**

	*x*	*y*	*z*	*U*_iso_*/*U*_eq_	
Br	0.96430 (19)	0.17863 (19)	0.67746 (8)	0.0634 (5)	
O	0.8640 (13)	0.2368 (15)	0.5056 (4)	0.062 (2)	
C1	1.0276 (16)	0.4425 (18)	0.6134 (6)	0.055 (3)	
H1A	0.9689	0.5875	0.6342	0.066*	
H1B	1.1661	0.4652	0.6138	0.066*	
C2	0.9577 (15)	0.4056 (11)	0.5267 (6)	0.039 (2)	

### Atomic displacement parameters (Å^2^)

**Table:**

	*U*^11^	*U*^22^	*U*^33^	*U*^12^	*U*^13^	*U*^23^
Br	0.0755 (7)	0.0501 (7)	0.0647 (9)	−0.0027 (7)	0.0041 (7)	0.0097 (6)
O	0.076 (5)	0.036 (3)	0.073 (6)	−0.026 (5)	−0.019 (5)	0.007 (3)
C1	0.045 (6)	0.068 (7)	0.052 (7)	0.000 (6)	0.009 (6)	−0.016 (7)
C2	0.033 (5)	0.028 (4)	0.057 (7)	0.013 (6)	0.023 (5)	0.007 (5)

### Geometric parameters (Å, º)

**Table:**

Br—C1	1.885 (10)	C1—H1A	0.9700
O—C2	1.196 (11)	C1—H1B	0.9700
C1—C2	1.585 (9)	C2—C2^i^	1.512 (16)
			
C2—C1—Br	112.4 (6)	H1A—C1—H1B	107.9
C2—C1—H1A	109.1	O—C2—C2^i^	124.6 (12)
Br—C1—H1A	109.1	O—C2—C1	123.7 (8)
C2—C1—H1B	109.1	C2^i^—C2—C1	111.4 (10)
Br—C1—H1B	109.1		
			
Br—C1—C2—O	4.7 (13)	Br—C1—C2—C2^i^	−169.0 (8)

Symmetry code: (i) −*x*+2, −*y*+1, −*z*+1.

## References

[bb1] Allen, F. H., Kennard, O., Watson, D. G., Brammer, L., Orpen, A. G. & Taylor, R. (1987). *J. Chem. Soc. Perkin Trans. 2*, pp. S1–19.

[bb2] Ducourant, B., Maury, C., Lere-Porte, J.-P., Petrissans, J. & Ribet, J.-L. (1986). *Acta Cryst.* C**42**, 341–343.

[bb3] Enraf–Nonius (1985). *CAD-4 Software* Enraf–Nonius, Delft, The Netherlands.

[bb4] Gogte, V. N., Shan, L. G., Tilak, B. D., Gadekar, K. N. & Sahasrabudhe, M. B. (1967). *Tetrahedron*, **23**, 2437–2441. 10.1016/0040-4020(67)80079-6 6044202

[bb5] Harms, K. & Wocadlo, S. (1995). *XCAD4* University of Marburg, Germany.

[bb6] North, A. C. T., Phillips, D. C. & Mathews, F. S. (1968). *Acta Cryst.* A**24**, 351–359.

[bb7] Ruggli, P. & Herzog, M. (1946). *Helv. Chim. Acta.*, 29, 95-101.

[bb8] Sheldrick, G. M. (2008). *Acta Cryst.* A**64**, 112–122. 10.1107/S0108767307043930 18156677

